# An Analysis of the Serum Metabolomic Profile for the Radiomitigative Effect of the Thrombopoietin Receptor Agonist Romiplostim in Lethally Whole-Body-Irradiated Mice

**DOI:** 10.3390/metabo12020161

**Published:** 2022-02-08

**Authors:** Yoshiaki Sato, Masaru Yamaguchi, Ikuo Kashiwakura

**Affiliations:** Department of Radiation Science, Hirosaki University Graduate School of Health Sciences, Hirosaki 036-8564, Aomori, Japan; h20gg702@hirosaki-u.ac.jp (Y.S.); masarun@hirosaki-u.ac.jp (M.Y.)

**Keywords:** metabolomics, thrombopoietin receptor agonist romiplostim, radiomitigative effect

## Abstract

The thrombopoietin receptor agonist romiplostim (RP) was recently approved by the US Food and Drug Administration for improving survival in patients acutely exposed to myelosuppressive doses of radiation. Our previous studies with mice have shown that RP administration after lethal irradiation not only completely rescues irradiated mice but also shows mitigative effects on their hematopoiesis and multiple organ injury, including that of the lung, bone marrow, small intestine, and liver. However, the mechanism by which RP functions as a radiomitigator remains unclear. In the present study, we applied a metabolomics approach, which has the ability to reflect the status of an organism directly and accurately, helping to elucidate the biology of treatment responses. Our results showed that the disruption of several metabolites and pathways in response to total body irradiation was partially corrected by RP administration. Notably, RP-corrected metabolites and pathways have been reported to be indicators of DNA damage and lung, bone marrow, small intestine, and liver injury. Taken together, the present findings suggested that the radiomitigative effect of RP is partially involved in the recovery of organ injury, and the identified metabolites may be a useful biomarker of the survival likelihood following radiation exposure.

## 1. Introduction

In the event of high-dose radiation exposure, death due to acute radiation syndrome (ARS), including myelosuppression and intestinal disorders, has been reported [1,2]. To address challenges associated with the discovery and development of medical countermeasures (MCMs) for ARS, the development of protective/mitigative agents against harmful effect of radiation has been ongoing for several decades [3,4].

Thus far, the US Food and Drug Administration (FDA) has approved to improve survival in patients acutely exposed to myelosuppressive doses of radiation, such as the granulocyte colony-stimulating factors (G-CSF) filgrastim (Neupogen^®^) and pegfilgrastim (Neulasta^®^) in 2015, and the granulocyte-macrophage colony-stimulating factor (GM-CSF) sargramostim (Leukine^®^) in 2018. These drugs are growth factors for neutrophils and hematopoietic progenitor cells [5]. 

In addition, a different type of MCM known as romiplostim (RP; Nplate^®^), a thrombopoietin (TPO) receptor (TPOR; myeloproliferative leukemia protein/CD110) agonist, was approved by the FDA in January 2021. TPO is a glycoprotein hormone, a hematopoietic factor involved in platelet production that promotes the proliferation and differentiation of megakaryocytes from hematopoietic progenitor cells in vivo [6]. Clinical trials of TPO for the treatment of thrombocytopenia have been carried out in several countries [7,8]. However, because of the discovery of induced antibodies that neutralize TPO and cause thrombocytopenia, its clinical development was stopped immediately [9]. The development of new types of TPO was subsequently continued, and at present, three drugs—romiplostim (RP), eltrombopag, and avatrombopag—are available as therapies for idiopathic thrombocytopenic purpura (ITP) [10,11,12]. Our previous studies showed that the RP completely rescued mice exposed to lethal total-body irradiation (TBI), suggesting that RP may not only promote hematopoiesis in various organs in irradiated individuals but also mitigate the dysfunction or regenerate the original function in multiple organs [13]. Previous studies have reported the following mechanisms by which RP exerts its radiation-mitigating effects on individuals exposed to radiation: restoration of the cell count in hematopoietic tissues, such as bone marrow and spleen; enhancement of DNA double-strand break repair and inhibition of apoptosis in hematopoietic cells [13]; and regulation of the expression of defense genes by the Nrf2-Keap1 system to regulate the expression of defense genes [14]. Although the function of RP in acute radiation injury is clear, the detailed mechanisms underlying how RP rescues mice exposed to lethal doses of radiation are still unknown.

Metabolomics is a post-genomics discipline that identifies and quantifies small molecules (50–1000 Da) located downstream of genomic, transcriptomic, and proteomic processes. The advantage of metabolomics is its ability to directly and accurately reflect the current status of organisms, helping to elucidate the biology of treatment responses [15]. Previously, metabolomics studies have been performed to identify molecular signatures of various pathological condition and therapies [16,17,18]. A growing number of studies have shown evidence that radiation exposure disrupts the biological pathway in various samples, such as serum and urine [19,20], providing a better understanding of the systemic effects of radiation exposure. However, there is little evidence as to what metabolic changes in exposed individuals should be targeted for mitigation of radiation damage.

In the present study, we conducted a capillary electrophoresis time-of-flight mass spectrometry (CE-TOFMS)-based metabolomic analysis to clarify the effect of RP on the metabolomics profile of serum derived from TBI and/or RP-treated mice.

## 2. Results

### 2.1. Effect of TBI on the Serum Metabolic Profile

In our previous report [13,21,22], lethally irradiated mice showed a decreased weight, but the weight of RP-treated lethally irradiated mice recovered relative soon after RP administration. As we suspected that there might be critical changes in RP-treated lethally irradiated mice that led to the mitigation of radiation injury, we performed CE-TOFMS-based metabolomics analyses of serum samples collected 7 days after TBI and/or RP treatment to understand the radiomitigative effect of RP. Based on the *m*/*z* values and migration times, 263 metabolites (162-cation and 101-anion mode) were identified (Supplementary file).

**Figure 1:**
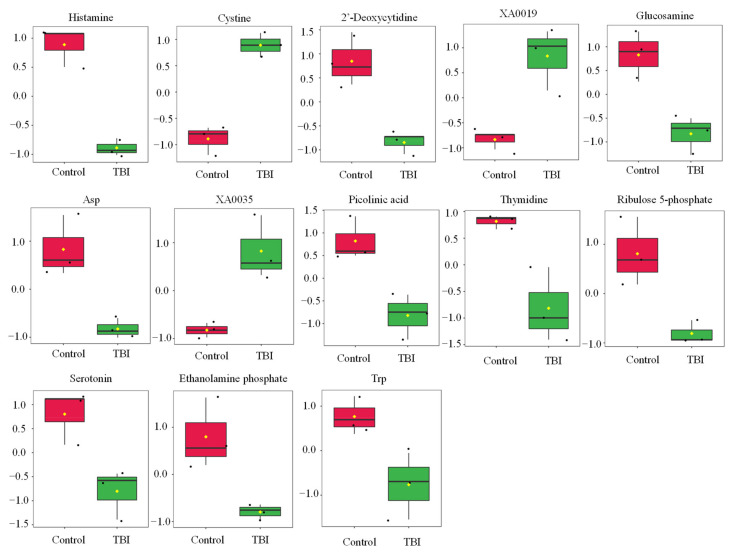
Effect of TBI on the metabolic profile in serum. Boxplots of metabolite were presented. The black dots represent the normalized value of metabolite. The notch indicates 95% confidence interval around the median. Asp—aspartate; Trp—tryptophan.

**Figure 2:**
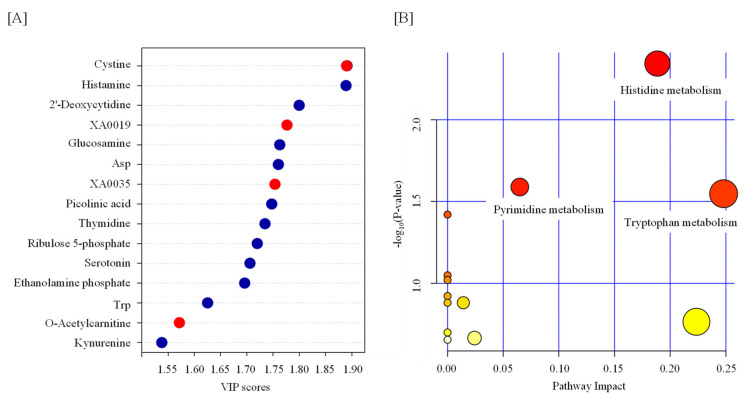
Feature of metabolite in the serum from TBI mice. (**A**) Variable importance in projection (VIP) scores of the partial-least-squares discriminant analysis (PLS-DA) in TBI and control mice. The red and blue dots indicate the corresponding metabolite level was increased and decreased by TBI, respectively. (**B**) A pathway analysis combining pathway enrichment and pathway topology analyses of metabolites whose levels were significantly changed by TBI. The *x*-axis marks the pathway impact, and the *y*-axis represents the pathway enrichment. Each node marks a pathway, with larger sizes and darker colors representing higher pathway impact values and greater pathway enrichment. Asp—aspartate; Trp—tryptophan.

Statistical tests revealed that the levels of 13 metabolites were significantly changed by TBI (Table 1 and Figure 1). We observed increases in cystine, XA0019, and XA0035 and decreases in histamine, 2′-deoxycytidine, glucosamine, aspartate (Asp), picolinic acid, thymidine, ribulose 5-phosphate, serotonin, ethanolamine phosphate, and tryptophan (Trp) in the serum of TBI mice (Table 1 and Figure 1). To identify the important features of the metabolomic profile in the serum of TBI mice, we performed a variable importance in projection (VIP) score analysis, allowing evaluating the importance of individual metabolites’ (from the predictors block) influence on the partial-least-squares regression model. Figure 2A shows that cystine and histamine were the most relevant metabolites. We further performed a pathway analysis with significantly altered metabolites to recognize the metabolic pathways that were disturbed due to TBI. A pathway analysis showed that three pathways (tryptophan metabolism; histidine metabolism; and pyrimidine metabolism) were significantly changed in response to TBI (Figure 2B and Table S1).

### 2.2. Effect of RP on the Serum Metabolic Profile

Although RP has been used for ITP [23], the effect of RP on the metabolomic profile remains unknown. Therefore, we investigated the impact in the present study. Statistical tests revealed that more metabolites in the serum of RP mice were affected than in the TBI mice. Overall, 26 metabolites were significantly changed upon RP treatment (Table S2 and Figure S1). We observed particularly significant increases in adenosine and guanosine in the serum of RP mice. We also observed that RP increased the levels of taurine, S-methylhistamine, and O-acetylcarnitine (Table S2 and Figure S1). In addition, we performed a VIP analysis and found that taurine was the most relevant metabolite (Figure S2A). A pathway analysis showed that five pathways (phenylalanine, tyrosine and tryptophan biosynthesis; taurine and hypotaurine metabolism; phenylalanine metabolism; arginine biosynthesis; and alanine, aspartate, and glutamate metabolism) were significantly changed upon RP treatment (Figure S2B and Table S3).

### 2.3. RP Attenuates Metabolic Changes in Response to TBI

**Figure 3:**
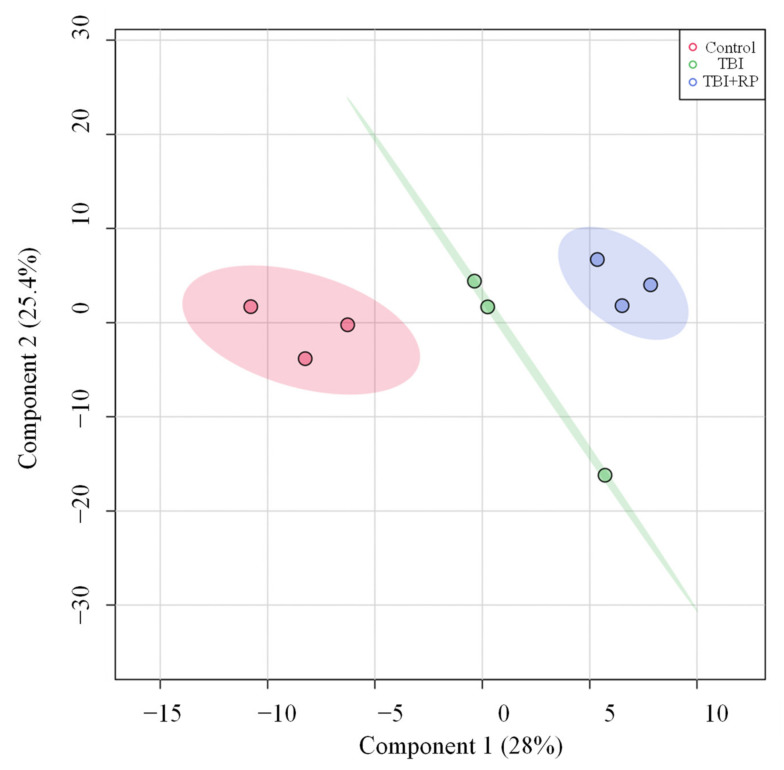
PLS-DA plots of the serum metabolome. PLS-DA plot shows separation for study groups control, TBI and TBI + RP based on metabolic profiles.

Given that RP administration resulted in the rescue of lethally irradiated mice [13,21,22], we investigated whether or not this effect was mediated by attenuation of TBI-induced metabolic profile perturbations. We used partial least square-discriminant analysis (PLS-DA) analysis to visualize group differences due to metabolic changes. PLS-DA algorithm includes predefined treatments/classes, and thus it can maximize the separation among treatment groups and maximize the covariance between metabolite variables to enable better understanding of the factors driving separation [24]. Group separation for changes can be seen (Figure 3). PLS-DA analysis showed three major clusters, where the first two components (one and two) explained 28 and 25.4% of the variance in the data set, respectively (Figure 3). 

**Figure 4:**
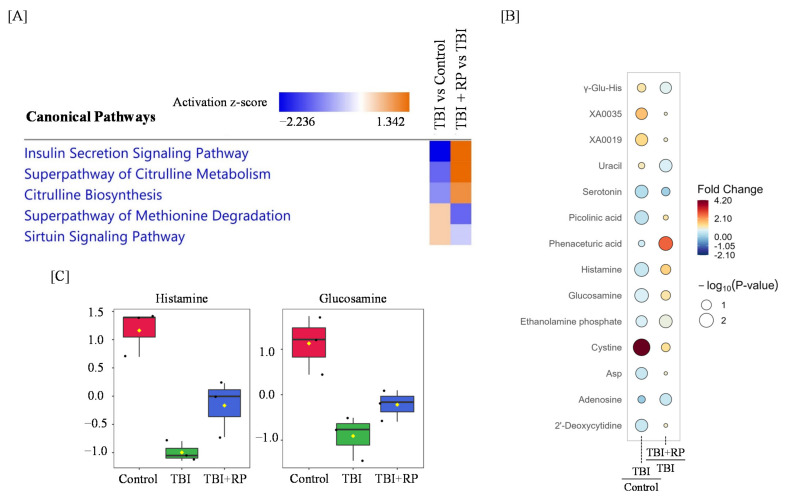
Radiomitigative effects of RP on the disruption of metabolome profiling and pathway by TBI. (**A**) An IPA analysis of the top canonical pathways in TBI and TBI + RP is represented. Colors indicate higher or lower Z scores. (**B**) Raindrop illustration of metabolite patterns showed fold changes between TBI and control or TBI + RP and TBI. The scale bar represents the normalized intensity of metabolites, where blue indicates a decrease/low and red indicates an increase/high. The size of the drop represents the −log_10_(*p*-value); Asp—Aspartate. (**C**) Boxplots of metabolite were presented. The black dots represent the normalized value of metabolite. The notch indicates 95% confidence interval around the median.

We performed a significant enrichment analysis of canonical pathways based on the ingenuity pathways analysis (IPA) database and found that the insulin secretion signaling pathway, superpathway of citrulline metabolism, citrulline biosynthesis, superpathway of methionine degradation, and sirtuin signaling pathway were dysregulated in response to TBI, while RP treatment following TBI restored these pathways (Figure 4A). Furthermore, a rain plot showed that some metabolites dysregulated by TBI, including histamine and glucosamine, were partially corrected by RP (Figure 4B,C). Interestingly, RP further increased the endogenous level of cystine, which had been increased by TBI (Figure 4B).

## 3. Discussion

We previously demonstrated that RP completely rescued mice exposed to lethal TBI [13,21,22]; however, the detailed mechanism by which RP rescued these mice has been unclear. To better understand the radiomitigative effect of RP, we applied a metabolomics approach using serum derived from TBI and/or RP-treated mice.

Most of the differentially expressed metabolites in response to TBI in this study have already been reported in previous radiation studies. For example, a decrease in the tryptophan and histidine metabolism, including tryptophan, serotonin, and histamine values, was reported in studies using mouse serum, mouse intestine, and rat urine [25,26,27,28,29]. These metabolites were reported to function as radioprotectors [30,31,32], and we observed that a decrease in these radioprotective metabolites helped explain the radiation-induced damage. We also observed a decrease in the components of the pyrimidine metabolism pathway, such as thymidine, in TBI mice, although several previous studies using mouse serum and urine showed that TBI upregulated the levels of thymidine, reflecting DNA or RNA damage [26,33]. This difference may have been due to the timing of sample collection following radiation exposure, since previous studies used samples collected at an earlier point (8–36 h) after radiation than the present study due to their aim of identifying radiation biomarkers.

Regarding our aim to investigate the effect of RP on metabolomic profile perturbation by TBI, the levels of histamine and glucosamine partially recovered following RP administration in TBI mice. Histamine, which was downregulated after TBI and reversed with RP in this study, reportedly protects radioresponsive tissues, such as the small intestine and bone marrow [30,34,35]. Medina et al., showed that histamine reduced radiation-induced toxicity by suppressing apoptosis of ductal and acinar cells [34], as well as the radiation-induced severe aplasia, edema, and vascular damage in the bone marrow of mice and rats [30]. Carabajal’s study in rats found that histamine pretreatment reduced ^137^Cs radiation-induced mucosal atrophy, edema, and vascular damage, increasing the number of crypts per circumference [35]. In addition, our previous reports showed that RP treatment recovered the decrease in the number of bone marrow cells and morphological integrity of the small intestines in irradiated mice [13,22], supporting our findings. We also observed a similar trend to glucosamine for histamine in the present study. Lei et al. reported that glucosamine alleviated ^60^Co radiation-induced lung injury, inhibited apoptosis, and promoted the proliferation of normal lung epithelial cells in vitro [36]. In addition, we previously showed that there was no marked difference in the number of viable lung cells in RP-treated mice, independent of TBI, although this value was decreased in irradiated mice without RP treatment [13]. Although we were unable to determine the concentration of histamine and glucosamine in serum, our findings suggest that they partially mediated the radiomitigative effect of RP on small intestine, bone marrow, and lung, thus contributing to the improvement of the survival of TBI mice. 

Interestingly, the increase in the level of cystine induced by TBI was further enhanced by RP. Cystine is well known to be associated with free-radical scavenging in response to radiation-induced oxidative damage [37,38]. Similar to our results, an increase in cysteine levels following X-ray irradiation in mouse serum was reported by Lee et al. [39], but no other report has described the effect of TPOR agonists on cysteine. Vlachodimitropoulou et al. showed that eltrombopag, a small-molecule oral TPOR agonist, reduced the reactive oxygen species in pancreatic cells [40]. Although why a combination of TBI + RP treatment remarkably increased cysteine levels is still unclear, this effect may have contributed to the countering effects of RP on radiation-induced oxidative damage.

We also performed a pathway analysis using IPA to clarify the effects of RP on mitigating the biochemical pathway perturbations caused by TBI. Insulin secretion, which is critical to human health, is regulated by pancreatic beta cells [41], and DNA damage induces beta cell failure that impairs insulin secretion [42]. Nylander et al., reported that insulin secretion was similarly dysregulated in skeletal muscle and adipose progenitor cells collected from irradiated mice [43]. Vlachodimitropoulou et al. showed that eltrombopag restored insulin secretion by pancreatic cells [40]. These reports supported our observation that the disruption of the insulin secretion signaling pathway by TBI was partially restored by RP, reflecting its mitigative effect on radiation-induced damage. Citrulline is a nitrogen end product of small bowel enterocyte glutamine metabolism and accounts for almost 30% of metabolized glutamine nitrogen in the small intestine [44]. Citrulline is reportedly associated with radiation-induced intestinal toxicity [45,46]. Jones et al. previously reported that citrulline was decreased at 3 days after TBI in mouse [47] and was characterized as a biomarker for gastrointestinal ARS [47,48,49]. Several studies have shown that citrulline levels positively correlate with the overall small bowel function [50,51]. These results may suggest that recovery of citrulline metabolism and biosynthesis reflect the mitigative effect of RP in the small intestine. 

We also observed an alleviating effect of RP on the disruption of the superpathway of methionine degradation in response to TBI (Figure 4A). Methionine plays a fundamental role in cell physiology, being involved in areas such as the methyl group supply, DNA methylation, and nucleotide biosynthesis, and changes in its dispensation have been reported to be associated with liver injury [52]. Liver injury is reportedly a harmful side effect of radiotherapy [53]. We previously showed that liver injury and protein expression associated with liver injury by TBI were mitigated by RP administration [13,54]. These reports suggest that the mitigating effect of RP on radiation-induced liver injury is mediated by the recovery of abnormalities in methionine metabolism. Notably, these injuries induced by IR have been reported to be recovered following RP administration in our previous studies. Although lethality was observed around day 7 after TBI in mice irradiated with lethal doses, limiting long-term studies, these critical changes in metabolite levels in response to a combination of TBI and RP presumably mediate the radiomitigative effect of RP on radiation injury.

Taken together, the present results showed that TBI induced disruption of several metabolites and pathways associated with DNA damage and oxidative stress as well as lung, bone marrow, and small intestine injury. Furthermore, RP administration following TBI partially recovered TBI-induced metabolic disruption. Although further studies are needed to address the role of identified metabolites in the mitigative effect of RP on radiation injury, the present results may provide a better understanding of the radiomitigative effect of RP and help improve the strategy for radioprotection/mitigation.

## 4. Materials and Methods

### 4.1. Ethics Statement

All experiments were conducted according to the legal regulations in Japan and the Guidelines for Animal Experiments after obtaining approval from the animal experimental committee (approved number: G17001), and all efforts were made to minimize the number of animals used and their suffering in this study. All mice were housed in standard cages in a conventional clean room under a 12-h light/dark cycle. The mice had ad libitum access to sterilized standard laboratory mouse chow diet (CLEA Rodent Diet CE-2, CLEA Japan) and drinking water.

### 4.2. Exposure of Mice to a Lethal Dose of X-ray

Seven-week-old female C57BL/6JJcl mice were delivered from the breeding facilities of Clea Japan (Tokyo, Japan). After acclimatizing for 1 week, 8-week-old mice were randomly subjected to 7 Gy of X-rays (150 kVp, 20 mA, 0.5-mm aluminum and 0.3-mm copper filters) at a dose rate of 1.0 Gy/min using an X-ray generator (MBR-1520R; Hitachi Medical Co., Tokyo, Japan) at a distance of 450 mm between the focus and the target. The air kerma was monitored with a thimble ionization chamber, which integrated the radiation dose and blocked X-rays when it reached a preset dose value.

### 4.3. Treatment with the Human Thrombopoietin-Mimetic c-mpl agonist RP

Within 2 h after TBI, mice were administered the human thrombopoietin-mimetic c-mpl agonist RP (Kyowa Hakko Kirin, Co., Ltd., Tokyo, Japan). In addition to mice that received TBI and RP (TBI + RP mice), mice treated with TBI only (TBI mice), those treated with RP only (RP mice), and those that received neither TBI nor RP (control mice) were also evaluated in this study. All groups consisted of three mice. RP was intraperitoneally administered once daily for 3 days, and the applied dose of RP was 50 µg/kg of body weight/day, which was determined based on the findings of previous reports [55,56,57]. Mice treated with TBI only and control mice alternatively received injections of normal saline solution (Otsuka Pharmaceutical, Tokyo, Japan) as the vehicle used to prepare the drug.

### 4.4. Sample Collection

Peripheral blood was harvested on day 7 after TBI from the orbital venous plexus of mice following anesthesia using isoflurane (Powerful Isoful; Zoetis, London, UK) by a capillary tube, and samples were left at room temperature for at least 30 min to allow for clotting. Serum was collected by centrifugation at 1200× *g* for 10 min and stored at −80 °C until the analysis. The stored samples were then transported packaged on dry ice from Hirosaki University in Aomori, Japan, to Human Metabolome Technologies Inc. (HMT) in Yamagata, Japan.

### 4.5. Metabolome Analyses

Metabolomics were performed through a facility service at HMT. In brief, 50 µL of serum was added to 200 µL of methanol containing internal standards (H3304-1002; HMT) at 0 °C to suppress enzymatic activity. The extract solution was thoroughly mixed with 150 µL of Milli-Q water, after which 300 µL of the mixture was centrifugally filtered through a Millipore 5-kDa cut-off filter (ULTRAFREE MC PLHCC; HMT) at 9100× *g*, 4 °C for 120 min to remove macromolecules. The filtrate was then evaporated to dryness under vacuum and reconstituted in 50 µL of Milli-Q water for the metabolome analysis at HMT.

The metabolome analysis was conducted according to HMT’s Basic Scan package, using CE-TOFMS based on the methods described previously [58,59]. In brief, CE-TOFMS analysis was carried out using an Agilent CE capillary electrophoresis system equipped with an Agilent 6210 time-of-flight mass spectrometer (Agilent Technologies, Inc., Santa Clara, CA, USA). The systems were controlled by Agilent G2201AA ChemStation software program, version B.03.01 (Agilent Technologies) and connected by a fused silica capillary (50 μm i.d. × 80 cm total length) with commercial electrophoresis buffer (H3301-1001 and I3302-1023 for cation and anion analyses, respectively; HMT) as the electrolyte. The spectrometer was scanned from *m*/*z* 50 to 1000, and peaks were extracted using the MasterHands, automatic integration software program (Keio University, Yamagata, Japan) to obtain peak information, including the *m*/*z*, peak area, and migration time (MT) [60]. Signal peaks corresponding to isotopomers, adduct ions, and other product ions of known metabolites were excluded, and the remaining peaks were annotated according to HMT’s metabolite database based on their *m*/*z* values and MTs.

### 4.6. Data Analyses Using Metaboanalyst and an IPA

Statistical and metabolic pathway analyses were carried out using the MetaboAnalyst 5.0 software package (http://www.metaboanalyst.ca/, Last accessed 3 February 2022), a comprehensive tool suite for metabolomics data analysis [61]. A part of data has missing value were removed when we analyzed. Auto-scaling was performed to normalize each compound. Two-sample *t*-tests was used to determine if there are differences in metabolite abundances. A pathway analysis based on KEGG (http://www.genome.jp/kegg/, Last accessed 3 February 2022) was performed to determine which pathways had been significantly perturbed. An IPA was performed with MetPA, a web-based tool for pathway analyses and visualization metabolomics. Statistical differences were considered significant when the test *p* value was <0.05.

## Figures and Tables

**Table 1:** List of TBI-induced metabolite dysregulation.

Name	Fold Change	*p* Value
cystine	4.2705	0.000991
histamine	0.4549	0.001072
2’-Deoxycytidine	0.5	0.007763
XA0019	1.3864	0.01047
glucosamine	0.68444	0.012282
Asp	0.5375	0.012634
XA0035	1.7021	0.013546
picolinic acid	0.39647	0.014431
thymidine	0.50685	0.016358
ribulose 5-phosphate	0.23725	0.01874
serotonin	0.28095	0.021134
ethanolamine phosphate	0.6748	0.022919
Trp	0.70548	0.037526

Probability means the *p* value determined by *t*-test. Data are represented as the ratio of peak area in TBI to that in control. Asp—aspartate; Trp—tryptophan.

## Data Availability

Data supporting reported results can be found in Supplementary file. xlsx: list of identified metabolites.

## Supplementary Materials

The following are available online at https://www.mdpi.com/article/10.3390/metabo12020161/s1, Supplementary file. xlsx: list of identified metabolites, Figure S1: Effect of RP on metabolic profile in serum, Figure S2: Feature of metabolite in the serum from RP mice; Table S1: TBI-induced metabolic pathway dysregulation, Table S2: List of RP-induced metabolite dysregulation, Table S3: List of RP-induced metabolic pathway dysregulation.
