# Supplementary material for: An Analysis of the Serum Metabolomic Profile for the Radiomitigative Effect of the Thrombopoietin Receptor Agonist Romiplostim in Lethally Whole-Body-Irradiated Mice
Source: Metabolites. 2022 Feb 8;12(2):161. doi: 10.3390/metabo12020161 (PMC8877426; doi:10.3390/metabo12020161)
Supplement: Supplementary file 1 [file metabolites-12-00161-s001.zip › metabolites-1575083-supplementary.pdf]

Supplementary Table S1. TBI-induced metabolic pathway dysregulation.

name	Total(Hit)	$-LOG_{10}(p)$	Impact	Expected
Histidine metabolism	16(2)	2.3435	0.18852	0.10624
Pyrimidine metabolism	39(2)	1.5884	0.06491	0.25896
Tryptophan metabolism	41(2)	1.5475	0.24798	0.27224
Aminoacyl-tRNA biosynthesis	48(2)	1.4198	0	0.31873
Arginine biosynthesis	14(1)	1.0485	0	0.092961
Nicotinate and nicotinamide metabolism	15(1)	1.0199	0	0.099602
Pantothenate and CoA biosynthesis	19(1)	0.9224	0	0.12616
beta-Alanine metabolism	21(1)	0.8815	0	0.13944
Sphingolipid metabolism	21(1)	0.8815	0.0142	0.13944
Alanine, aspartate and glutamate metabolism	28(1)	0.7655	0.22356	0.18592
Cysteine and methionine metabolism	33(1)	0.7006	0	0.21912
Glycerophospholipid metabolism	36(1)	0.6666	0.02423	0.23904
Amino sugar and nucleotide sugar metabolism	37(1)	0.6560	0	0.24568

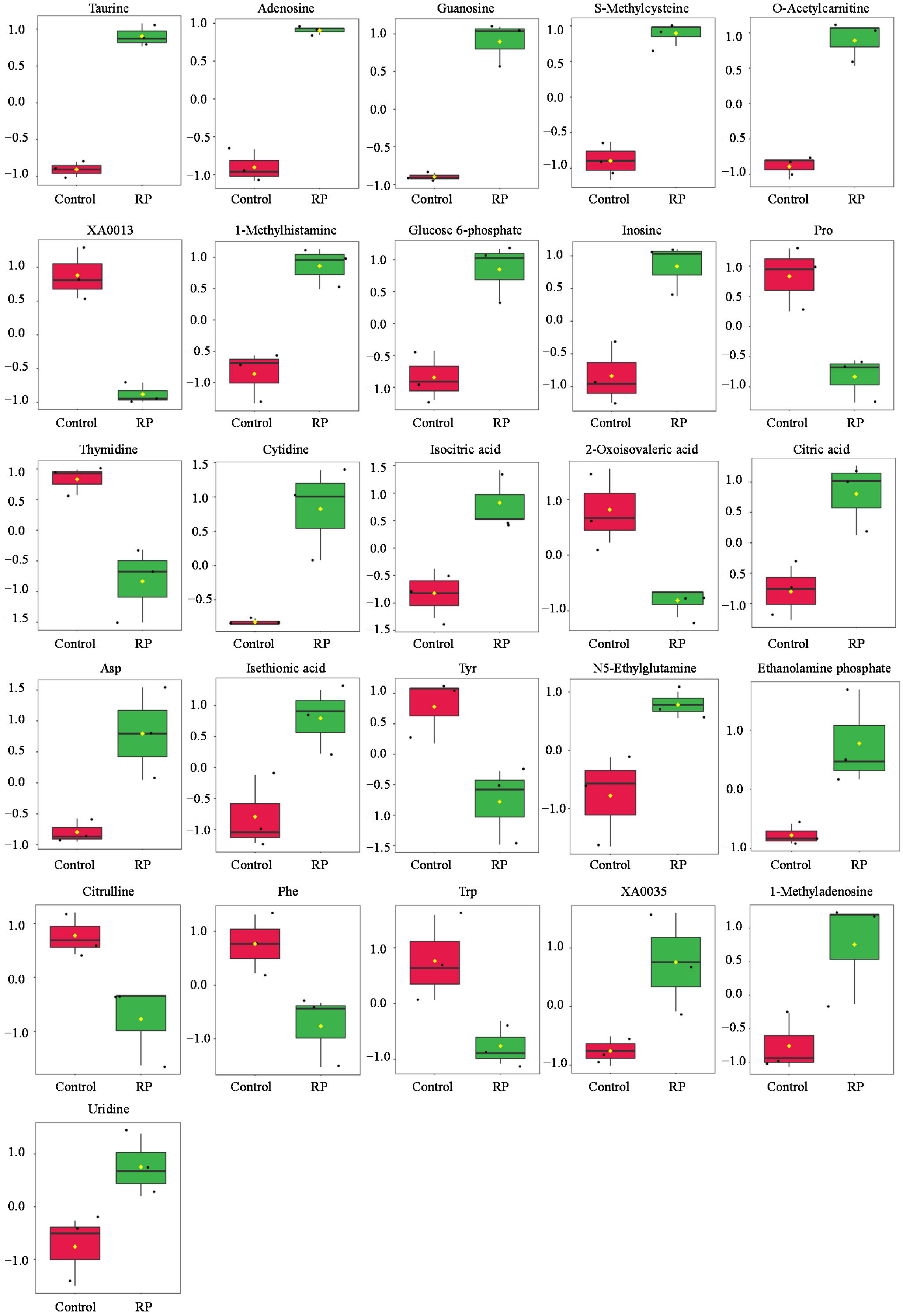
Taurine
Adenosine
Guanosine
S-Methylcysteine
O-Acetylcarnitine
XA0013
1-Methylhistamine
Glucose 6-phosphate
Inosine
Pro
Thymidine
Cytidine
Isocitric acid
2-Oxoisovaleric acid
Citric acid
Asp
Isethionic acid
Tyr
N5-Ethylglutamine
Ethanolamine phosphate
Citrulline
Phe
Trp
XA0035
1-Methyladenosine
Uridine
Control
RP

Supplementary Figure S1 Effect of RP on the metabolic profile in serum. Boxplots of metabolite were presented. The black dots represent the normalized value of metabolite. The notch indicates 95% confidence interval around the median. Proline, Pro; Aspartate, Asp; Tyrosine, Tyr; Phenylalanine, Phe; Tryptophan, Trp.

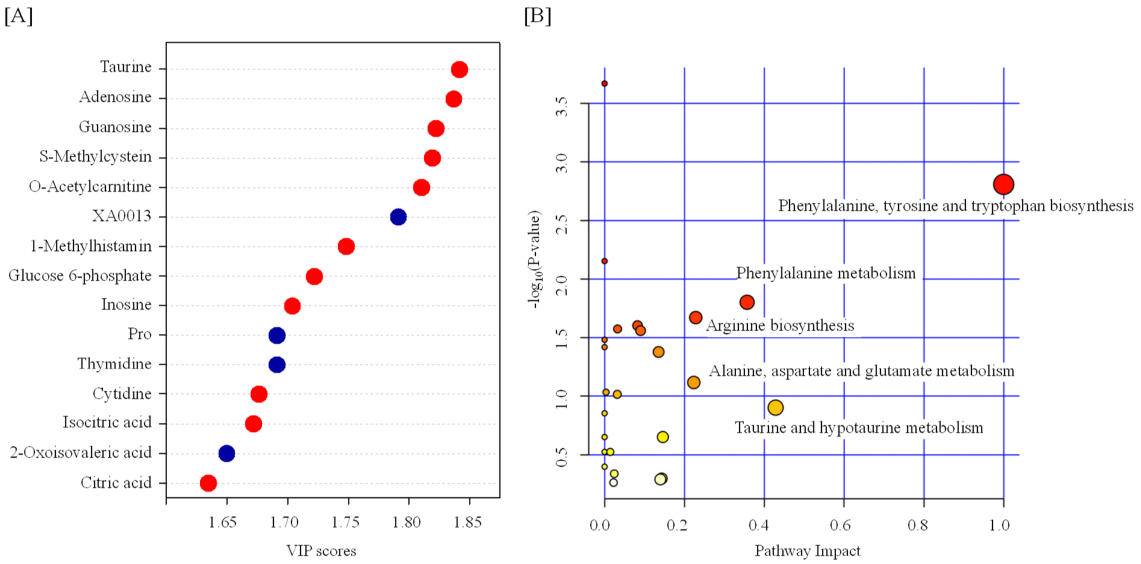

Supplementary Figure S2. Feature of metabolite in the serum from RP mice. [A] Variable Importance in Projection (VIP) scores of the partial least squares discriminant analysis (PLS-DA) in RP and control mice. The red and blue dots indicate the corresponding metabolite level was increased and decreased by RP, respectively. Proline, Pro. [B] A pathway analysis combining pathway enrichment and pathway topology analyses of metabolites whose levels were significantly changed by RP. The x-axis marks the pathway impact, and the y-axis represents the pathway enrichment. Each node marks a pathway, with larger sizes and darker colors representing higher pathway impact values and greater pathway enrichment.

Supplementary Table S2. List of RP-induced metabolite dysregulation.

name	Fold Change	p.value
Taurine	1.963	8.01E-05
Adenosine	10.966	0.000143
Guanosine	14.203	0.000462
S-Methylcysteine	1.4762	0.000562
O-Acetylcarnitine	1.4255	0.000864
XA0013	0.1097	0.001752
1-Methylhistamine	2.5439	0.004885
Glucose 6-phosphate	2.3291	0.007551
Inosine	2.3113	0.009707
Pro	0.69504	0.011377
Thymidine	0.71233	0.011392
Cytidine	1.3316	0.013477
Isocitric acid	1.2157	0.014173
2-Oxoisovaleric acid	0.82812	0.017704
Citric acid	1.2452	0.020364
Asp	2.4375	0.023424
Isethionic acid	1.5768	0.025191
Tyr	0.73504	0.029804
N5-Ethylglutamine	1.6265	0.029831
Ethanolamine phosphate	2.3659	0.031122
Citrulline	0.75342	0.033472
Phe	0.73077	0.036946
Trp	0.83562	0.038103
XA0035	1.383	0.04049
1-Methyladenosine	1.4658	0.04112
Uridine	1.5854	0.041214

Probability means the P value determined by t-test. Data are represented as the ratio of peak area in RP to that in control. Proline, Pro; Aspartate, Asp; Tyrosine, Tyr; Phenylalanine, Phe; Tryptophan, Trp.

Supplementary Table S3. List of RP-induced metabolic pathway dysregulation.

name	Total(Hit)	$-LOG_{10}(p)$	Impact	Expected
Phenylalanine, tyrosine and tryptophan biosynthesis	4(2)	2.8812	1	0.061089
Aminoacyl-tRNA biosynthesis	48(4)	2.2879	0	0.73307
Valine, leucine and isoleucine biosynthesis	8(2)	2.2284	0	0.12218
Phenylalanine metabolism	12(2)	1.8722	0.35714	0.18327
Arginine biosynthesis	14(2)	1.7407	0.22843	0.21381
Pyrimidine metabolism	39(3)	1.7017	0.0825	0.59562
Histidine metabolism	16(2)	1.6286	0.09016	0.24436
Neomycin, kanamycin and gentamicin biosynthesis	2(1)	1.5183	0	0.030544
Pantothenate and CoA biosynthesis	19(2)	1.4869	0	0.29017
Citrate cycle (TCA cycle)	20(2)	1.4451	0.13536	0.30544
Alanine, aspartate and glutamate metabolism	28(2)	1.1784	0.22356	0.42762
Purine metabolism	66(3)	1.1185	0.00366	1.008
Glyoxylate and dicarboxylate metabolism	32(2)	1.0763	0.03175	0.48871
Taurine and hypotaurine metabolism	8(1)	0.93517	0.42857	0.12218
Valine, leucine and isoleucine degradation	40(2)	0.91136	0.02168	0.61089
Ubiquinone and other terpenoid-quinone biosynthesis	9(1)	0.88716	0	0.13745
Nicotinate and nicotinamide metabolism	15(1)	0.68402	0	0.22908
Starch and sucrose metabolism	15(1)	0.68402	0.14607	0.22908
beta-Alanine metabolism	21(1)	0.55636	0	0.32072
Sphingolipid metabolism	21(1)	0.55636	0.0142	0.32072
Inositol phosphate metabolism	30(1)	0.42871	0	0.45817
Glycerophospholipid metabolism	36(1)	0.36739	0.02423	0.5498
Tryptophan metabolism	41(1)	0.3256	0.14305	0.62616
Tyrosine metabolism	42(1)	0.31806	0.13972	0.64143
Primary bile acid biosynthesis	46(1)	0.29016	0.02239	0.70252
